# The International Society of Surgeons

**Published:** 1905-11

**Authors:** Edgar R. McGuire

**Affiliations:** Buffalo, N. Y., 1175 Main Street


### SPECIAL ARTICLE.

---

### The International Society of Surgeons.

By EDGAR R. McGUIRE, M. D., Buffalo, N. Y.

IN COMPANY with Dr. Roswell Park of this city, I was privileged to attend the first congress of the International Society of Surgeons at Brussels on September 18, 1905. The fact that the society is composed of six hundred and fifty of the foremost surgeons of the world, about one-half of whom were present, makes the meeting most interesting and its transactions exceedingly valuable.

For several years American surgeons have felt the need of establishing some means by which surgeons of different nations could meet to discuss important surgical subjects. A few years ago Dr. Keen, of Philadelphia, introduced the subject to the American Surgical Association. The matter was favorably received and some correspondence was attempted in its behalf. But the time was, evidently, not ripe for such a movement and the matter went by default until three years ago, when the Belgian Society of Surgeons took it up. Dr. Willems (Gand), president of the Belgian Society, was made president of a Belgian and International Committee, which was instructed to arrange for the organisation of an international society. The recent meeting at Brussels was the result of the committee's work.

The International Committee was formed by choosing one man from each country. Dr. Roswell Park, of Buffalo, was the delegate from the United States. Professor Kocher, Bern, was elected president. The committee had charge of the executive work of the congress.

Its constitution is very simple. Each country is represented by a committee of three, the chairman to be delegate to the congress and a member of the International Executive Committee. Meetings are held every three years when members are assessed eight dollars. Every meeting is open to the profession. The official languages are French, German and English.

The first congress was opened September 18, by addresses of welcome by the Belgian minister of agriculture, representing the King, the surgeon-general of the Belgian army, Dr. Depage of Brussels, and Prof. Kocher. In the adjoining rooms was an exceedingly interesting exhibition of surgical instruments, apparatus and supplies.

The pian followed by the congress was to devote the mornings to either visiting the hospitals where interesting clinics were given by the Brussels surgeons, or the anatomical institute where demonstrations were given on the cadaver. These proved an exceedingly valuable part of the meeting. The afternoons were given up to papers and their discussion.

Monday, the first day, "The value of examinations of the blood in surgery," was discussed. Papers in discussion were read by Ortiz De La Torre, Madrid; Sonnenberg, Berlin; Keen, Philadelphia, and Depage, Brussels. The subject was particularly well handled by Dr. Keen. He gave an elaborate review, taking up in detail, coagulating time, cryoscopy iodophilia, hemoglobin, and leukocytosis. He maintained that blood examinations had been proven to be of value; but one should not make the mistake of placing too much confidence in the result of any examination, but should rather consider each in common with other signs and symptoms. He strongly recommended more systematic study of the blood so that the future might give us more positive knowledge.

Tuesday, the discussion was upon hypertrophy of the prostate and the diagnoses of surgical affections of the kidney. Papers were read by Rydygier, Lemberg; Harrison, London; Rovsing, Copenhagen; Albarran, Paris; Kummel, Hamburg; and Giordano, Venice. A very lively discussion took place. While most Americans favor the perineal operation, Europeans, as a rule favor the suprapubic route. In fact many of the men stated that while they formerly did the perineal operation, they now invariably do the suprapubic. It is evidently a very difficult question to settle but nowhere could the value of such a congress be better demonstrated than in a discussion of this important topic.

The subject on Wednesday was the treatment of non-cancerous affections of the stomach. The varied views on this topic could not fail to bring a spirited discussion. Papers were read by Monprofit, Angers; Rotgans, Amsterdam; Mayo Robson, London; Mattoli, Ascoli Piceno; von Eiselsburg, Vienna; and Jonnesco, Bucharest. The discussion was participated in by such men as Kocher, Czerny, Hartmann, and Legrand.

Posterior gastroenterostomy with short loop, seemed to be in most favor. Some held to the Murphy button while others favored a complete resection of the ulcer-bearing area.

On Thursday, the treatment of tubercular arthritis was discussed. Papers were submitted by Bier. Bonn; Broca, Paris; Willems, Gand; Coderilla, Bologna; and Bradford, Boston. Biers's paper offered further evidence as to the value of his treatment by passive congestion. Bradford's paper was very highly recommended, reflecting due honor upon America.

Friday was given up to the treatment of peritonitis. Papers were presented by Frederick, Berlin; Krogins, Helsingfors; Lennander, Upsala; Legar, Paris; De Isla, Madrid; and McCosh, New York. Dr. McCosh read a very valuable paper. He advised immediate operation in the majority of cases, believing, however, a few were better treated by starvation and absolute intestinal rest. Rapid operation, gentleness and Fowler's position were deciding factors. He spoke against irritating irrigations.

At the General Assembly on Friday afternoon, Professor

Czerny was elected president and Dr. Park was re-elected delegate from the United States.

Cancer was chosen as one topic for the next congress which will again be held at Brussels.

Brussels is a very beautiful city, centrally located, hence the choice was a popular one. The pleasure of the meeting was very greatly enhanced by the delightful entertainments given by the Belgians. Dinners and receptions were given each evening. Wednesday evening all the members and visitors attended the grand opera as the guests of the Belgian Society. On Thursday evening a general banquet was given in the Chamber of Commerce building. Short speeches were made by representatives from the leading nations.

Dr. Park in responding for America proposed three American cheers and it is doubtful if the historic old place ever witnessed so enthusiastic a scene. The Americans were in good voice. Altogether everyone enjoyed a most delightful and profitable meeting and came away feeling that the first International Congress was a brilliant success.

The great hope of American surgery lies in the fact that our surgeons are keeping in touch with the work of the men of other nations. This cannot be said to the same degree of any other country.

The American members present were Drs. Binnie, Kansas City; Bradford, Mixter, Richardson, Warren, and Cabot, Boston; McCosh and Dennis, New York; Freeman, Denver; Gerrish, Portland, Me.; Hingston, Montreal; Keen, Philadelphia; Lutz, St. Louis; McArthur, Chicago; and Park, Buffalo; there were also five others, Wills, LosAngeles; Tait, SanFrancisco; Yeomans, New York; and Max. Breuer and McGuire, Buffalo.

During our stay in Paris occurred the tenth anniversary of Pasteur's death. His tomb lies in a prominent place in the Pasteur Institute. It is beautiful, yet simple. What a peculiar trait in human character that the French people today should kneel so reverently at the tomb of the great Napoleon, while the greatest of all Frenchmen, Pasteur, should lie unnoticed except for the few men of science who are drawn to pay tribute to his memory. In the Pasteur Institute, however, he has a monument worthy of his name. It is one of the rewards of science, where men give their life to the study of disease with no recompense save that which comes from duty well done.

While waiting for our steamer at Boulogne we saw a monument to Edward Jenner. In England, his home, art galleries are filled with monuments to Nelson, Wellington, and other military heroes, but nowhere do you find a memorial to the man who rid Europe of that awful scourge,—smallpox. Yet in this small seaport town of France is a fitting remembrance erected by its citizens in recognition of his services to mankind.

1175 MAIN STREET.

